# Production of ^211^At and automated radiosynthesis of [^211^At]MABG via electrophilic astatodesilylation

**DOI:** 10.1186/s41181-025-00376-1

**Published:** 2025-08-05

**Authors:** Yuto Kondo, Taiki Joho, Shigenori Sasaki, Kazumasa Mochizuki, Naoko Hasegawa, Naoyuki Ukon, Ken-ichi Nishijima, Kohshin Washiyama, Hiroshi Tanaka, Tatsuya Higashi, Noriko S. Ishioka, Kazuhiro Takahashi

**Affiliations:** 1 Advanced Clinical Research Center, Fukushima Global Medical Science Center, Fukushima Medical University, 1 Hikarigaoka, Fukushima, 960-1295 Japan; 2 SHI Accelerator Service Ltd., 7-1-1 Nishigotanda, Shinagawa, Tokyo 141-0031 Japan; 3 Department of Chemical Science and Engineering, Institute of Science Tokyo, 12-12-1-H101 Ookayama, Meguro, Tokyo 152-8552 Japan; 4 Laboratory of Pharmaceutical Chemistry, Juntendo University, 6-8-1 Hinode, Urayasu, Chiba 279-0013 Japan; 5 Department of Molecular Imaging and Theranostics, Institute for Quantum Medical Science, National Institutes for Quantum Science and Technology, 4-9-1, Anagawa, Inage, Chiba-City, Chiba 263-8555 Japan; 6 Department of Quantum-Applied Biosciences, Takasaki Institute for Advanced Quantum Science, National Institutes for Quantum Science and Technology, 1233 Watanuki, Takasaki, Gunma 370-1292 Japan

**Keywords:** Astatine-211, MABG, Dry distillation, Automated synthesizer, Astatodesilylation, Radiochemistry, TAT

## Abstract

**Background:**

[^211^At]*m*-Astatobenzylguanidine ([^211^At]MABG) has demonstrated potent antitumor efficacy in preclinical models of malignant neuroendocrine tumours including neuroblastoma and pheochromocytoma/paraganglioma. The high linear energy transfer and short tissue penetration range of alpha particles enable highly localized cytotoxic effects, potentially overcoming therapeutic limitations associated with conventional beta-emitting radiopharmaceuticals. However, under clinical-scale (i.e., high radioactivity) conditions, the efficient and stable production of [^211^At]MABG has been hindered by radiolytic degradation during the manufacturing process limiting the availability of reliable methods offering high radiochemical yield and purity. In this study, we aimed to develop a scalable production methodology for [^211^At]MABG suitable for clinical translation.

**Results:**

^211^At was produced via the ^209^Bi(α,2n)^211^At nuclear reaction using a cyclotron, with ^210^At formation minimised by precise control of the alpha particle energy. The resulting product was purified using an automated dry distillation system. [^211^At]MABG was synthesised using the COSMiC-Mini automated synthesiser in 28.2 ± 2.8 min from initial ^211^At activities of up to 586.1 MBq. The radiochemical yield and purity were 80.3 ± 4.4% (decay-corrected RCY: 84.0 ± 4.5%) and 99.0 ± 0.7%, respectively (n = 6). The addition of sodium ascorbate as a radical scavenger contributed to maintaining a high radiochemical yield and purity during large-scale production. The final product was obtained as a sterile solution.

**Conclusions:**

In this study, we established a reliable and scalable production methodology for [^211^At]MABG, consistently achieving high radiochemical yield and purity across a wide range of radioactivity levels through optimization of the automated radiosynthesis process and the use of radiolytic stabilizers. This approach provides a solid technical foundation for the clinical application of [^211^At]MABG in targeted alpha therapy.

**Supplementary Information:**

The online version contains supplementary material available at 10.1186/s41181-025-00376-1.

## Background

The norepinephrine transporter is overexpressed in neuroendocrine tumours (NETs), including neuroblastoma, pheochromocytoma/paraganglioma, and carcinoid tumours, making it an attractive target for the selective delivery of therapeutic radiopharmaceuticals. [^123^I]MIBG and [^131^I]MIBG are among the widely used radiopharmaceuticals for NETs, offering dual functionality for both disease imaging and targeted radionuclide therapy. Despite their clinical benefits, limitations in their therapeutic efficacy—particularly in refractory or advanced disease—have prompted the search for alternative radionuclides to improve outcomes (Streby et al. 2015; Pandit-Taskar and Modak 2017; Pryma et al. 2019).

Among alternative therapeutic agents, [^211^At]MABG has emerged as a promising candidate (Cunningham et al. 1998; Ohshima et al. 2018, 2019). The α-particles emitted by ^211^At exhibit high linear energy transfer (LET) and a short path length in tissue, resulting in highly localized cytotoxic effects (Baidoo et al. 2013). Theoretically, [^211^At]MABG is expected to offer superior therapeutic efficacy, a notion supported by preclinical studies demonstrating pronounced antitumor activity against malignant pheochromocytoma and neuroblastoma (Cunningham et al. 1998; Ohshima et al. 2018, 2019; Baidoo et al. 2013). In addition to its therapeutic advantages, [^211^At]MABG offers notable improvements in radiation safety and patient quality of life (QOL). Unlike [^131^I]MIBG, which emits γ-rays over an extended period (^131^I half-life: 8.0 d) requiring strict isolation protocols and contact restrictions, [^211^At]MABG benefits from the shorter half-life of ^211^At (7.2 h) and the limited penetration range of α-particles, allowing for reduced isolation periods and significantly lower radiation exposure to those in proximity (Kobayakawa et al. 2024). Thus, [^211^At]MABG offers significant advantages in clinical management. From the perspectives of both therapeutic efficacy and radiation safety, further studies aimed at the clinical application of [^211^At]MABG are therefore of considerable importance.

Radiosynthetic methods for [^211^At]MABG have been extensively studied by Zalutsky et al. (Vaidyanathan and Zalutsky 1992; Vaidyanathan et al. 2007a, 2007b) Approaches employing C_18_ solid-phase extraction (SPE) columns in the purification process were prone to radiolytic degradation during the concentration steps. Notably, the use of ion exchange columns eliminated the need for these concentration steps, enabling the production of [^211^At]MABG at large scales (^211^At: 14–658 MBq) with an RCY of 63 ± 9% and RCP exceeding 90% (Vaidyanathan et al. 2007b). However, residual radio-byproducts (~ 10%) and variability in RCY remained unresolved, indicating that radiolytic degradation during the manufacturing process was not completely suppressed, possibly hindering further scale-up. Furthermore, the use of organotin precursors introduces the risk of residual tin in the final [^211^At]MABG solution, requiring additional post-synthetic quality control analyses via inductively coupled plasma mass spectrometry (ICP-MS). These findings highlight the persistent challenges in establishing reliable and scalable radiosynthesis protocols suitable for clinical application. More recently, alternative astatination strategies that avoid the use of tin precursors—such as those employing iodonium salts (Guerard et al. 2016, 2017; Navarro et al. 2019; Matsuoka et al. 2021; Maingueneau et al. 2022) or aryl boronic acids (Reilly et al. 2018; Watanabe et al. 2024)—have gained increasing attention, offering diverse radiolabeling routes. However, scalable methods for large-scale production of [^211^At]MABG remain limited. Therefore, to address these challenges, we aimed to develop a robust and scalable production methodology for [^211^At]MABG, optimised for clinical translation.

## Methods

### Reagents and instrumentation

Reagents used for synthesis and analysis were purchased from Fujifilm Wako Chemical Corp. (Osaka, Japan) and Tokyo Chemical Industry Co., Ltd. (Tokyo, Japan). Unless otherwise indicated, these reagents were used without additional purification. 1-(3-(Trimethylsilyl)benzyl)guanidine (*m*TMSBG) was purchased from ABX (Radeberg, Germany). Bismuth (6N) was purchased from Goodfellow Cambridge Ltd. (Huntingdon, England).

γ-Ray spectrometry was performed using a high-purity germanium (HP-Ge) detector (ORTEC GEM30-70) coupled to a multichannel analyser (Seiko EG&G MCA-7). Spectra were acquired with the sample placed at a distance of 50 cm from the detector. For measurements performed after the purification of ^211^At, an 8-mm thick lead shield was inserted between the sample and the detector to reduce dead time and radiation damage.

Radioactivity measurements were performed using a CRC-25R dose calibrator (Capintec Inc., Mirion Technologies, Inc. NJ, USA).

High performance liquid chromatography (HPLC) analyses were performed using an LC-20AB system (Shimadzu, Kyoto, Japan) equipped with a CBM-20A communication bus module, DGU-20A3R degassing unit, CTO-20AC column oven, SPD-M20A ultraviolet (UV) detector (all Shimadzu), and GABI Star radioactivity detector (Elysia-raytest GmbH, Straubenhardt, Germany). Reverse-phase HPLC was carried out on a YMC-Triart C_18_ column (4.6 mm I.D. × 300 mm; YMC CO., LTD.). Data acquisition and processing were performed using LabSolutions software (Shimadzu). HPLC analysis was performed using a YMC-Triart C_18_ column (4.6 mm I.D. × 300 mm) with gradient elution. The mobile phases were: solvent A, acetonitrile containing 0.1% (v/v) trifluoroacetic acid (TFA); and solvent B, water containing 0.1% (v/v) TFA. The gradient program was as follows: 0–20 min, 30–70% A; 20–21 min, 70–100% A; 21–30 min, 100% A; 30–35 min, 100–30% A; 35–50 min, 30% A. The flow rate was set at 1.0 mL/min, the column temperature was maintained at 25 °C, and the injection volume was 100 µL. HPLC analysis of TFA was performed using a YMC-Pack ODS-AQ (4.6 I.D. × 150 mm) with isocratic elution using 50 mM NaH_2_PO_4_ (0–15 min) as the mobile phase. The flow rate was 0.5 mL/min, the column temperature was maintained at 30 °C, and the injection volume was 5 µL.

Gas chromatography (GC) analyses were performed using a Shimadzu GC-2010 PlusAF system equipped with a flame ionization detector (FID). Separations were carried out on an Inertcap 624 column (30 m × 0.53 mm I.D., 3.0 µm film thickness; GL Sciences Inc.). Helium (99.999%) was used as the carrier gas at a constant flow rate of 0.5 mL/min. Samples (0.5 µL) were injected in split mode at an injector temperature of 240 °C. The oven temperature was initially set at 40 °C (held for 7 min), ramped to 120 °C at a rate of 20 °C/min (held for 1 min), and decreased to 40 °C at a rate of − 30 °C/min (held for 3 min). The FID temperature was maintained at 240 °C. Data acquisition and processing were performed using LabSolutions software (Shimadzu).

### Production of ^211^At

^211^At was produced on MP-30 (Sumitomo Heavy Industries, Tokyo, Japan) via the ^209^Bi(α,2n)^211^At reaction in the Advanced Clinical Research Center of Fukushima Medical University. The ^209^Bi target plate was produced via physical vapor deposition using the RD-1230 (SANVAC Co. Ltd., Tokyo, Japan). High-purity ^209^Bi (purity: 99.9999%, 2 mg) was vaporized under vacuum pressure to form the ^209^Bi layer (0.09 mm) on an aluminium base (Φ = 23 mm, l = 4 mm).

**Fig. 1:**
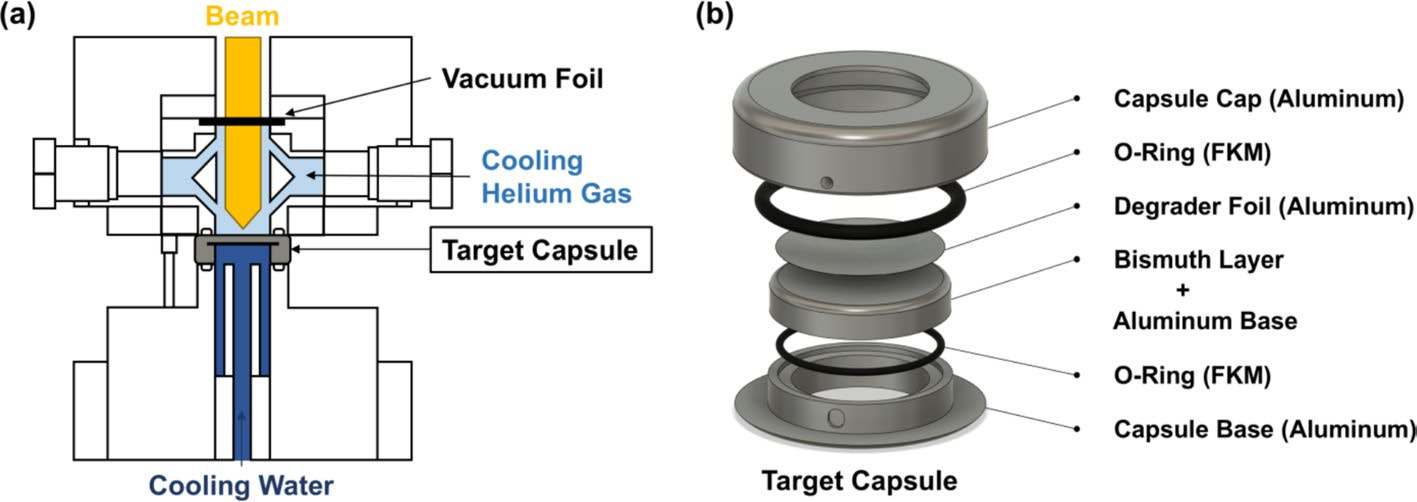
**a** Schematic cross section of the irradiation port; **b** 3D schematic of the target capsule

For irradiation, the ^209^Bi plate was mounted in a target holder, and alpha particles with an initial energy of 31.6 MeV were directed perpendicularly to the target surface (Fig. 1). During irradiation, the target was cooled using helium gas and water. An aluminium degrader foil (thickness: 35 µm) was positioned upstream of the target to reduce the particle energy at the target surface to 28.6 MeV. The beam current was typically maintained at 20 µA, with irradiation durations ranging from 1 to 3 h depending on the desired yield. The thick target yields (TTYs, expressed as MBq/μA·h) were determined from the measured activity of ^211^At at the end of bombardment (EOB), beam current, and irradiation time.

Following irradiation, the production of ^211^At was confirmed via γ-ray spectrometry using an HP-Ge detector (ORTEC GEM30-70) coupled to a multichannel analyser (Seiko EG&G MCA-7). The activated target was subsequently transferred to a dry distillation system for ^211^At separation and purification.

### Separation and purification of ^211^At

**Fig. 2:**
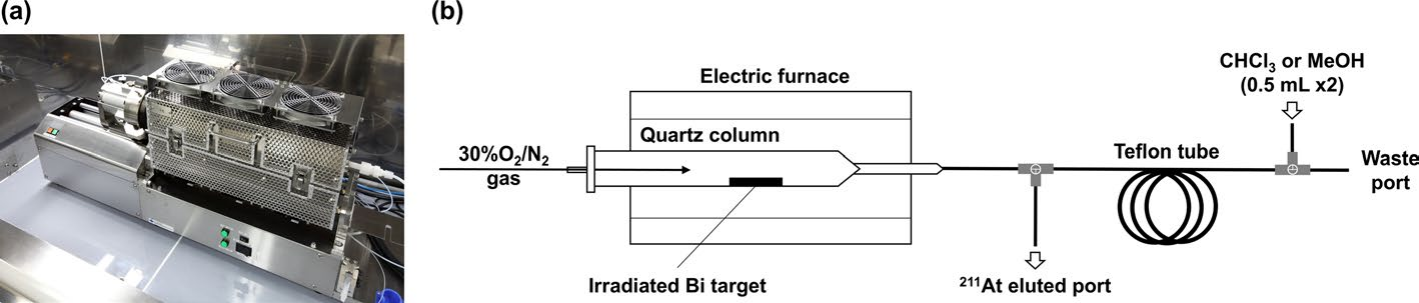
**a** Photograph of the At-HDS100 dry distillation system; **b** Schematic of the dry distillation system for the purification of ^211^At

^211^At was separated and isolated from the irradiated ^209^Bi target via dry distillation using the automated dry distillation system At-HDS100 (Sumitomo Heavy Industries, Ltd, Tokyo, Japan) installed in a hot cell (Fig. 2). Prior to distillation, one of the electric furnace heaters was preheated to 800 °C. The irradiated ^209^Bi target was then promptly placed inside the quartz column and heated to 850 °C under a continuous flow of 30% O_2_/N_2_ gas (40 mL/min). The gas stream was introduced into a Teflon tube (2 mm I.D. × 3 mm O.D., 50 cm), and its activity was monitored using a γ-ray spectrometer GR1-A (Kromek, County Durham, UK). Once the radioactivity of the irradiated target reached a plateau, the gas supply and heating were discontinued. The trapped ^211^At was eluted from the Teflon tube using either chloroform (CHCl_3_; 500 µL × 2) or methanol (MeOH; 500 µL × 2) and collected into a glass vial. The radioactivity and radionuclidic purity were measured and evaluated using a dose calibrator and an HP-Ge detector connected to a multichannel analyser, respectively.

### Automatic synthesis of [^211^At]MABG

**Scheme 1:**
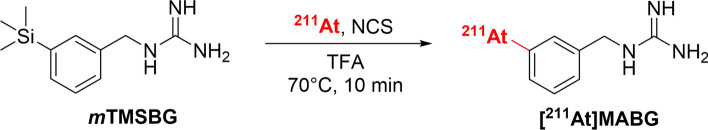
Synthesis of [^211^At]MABG using an aryl trimethylsilyl precursor. Reaction conditions: *m*TMSBG (1 μmol); NCS (2 μmol); solvent, TFA (0.5 mL); temperature, 70 ˚C; time, 10 min. *K_2_CO_3_ (72 μmol) was added upon using MeOH to elute ^211^At

**Fig. 3:**
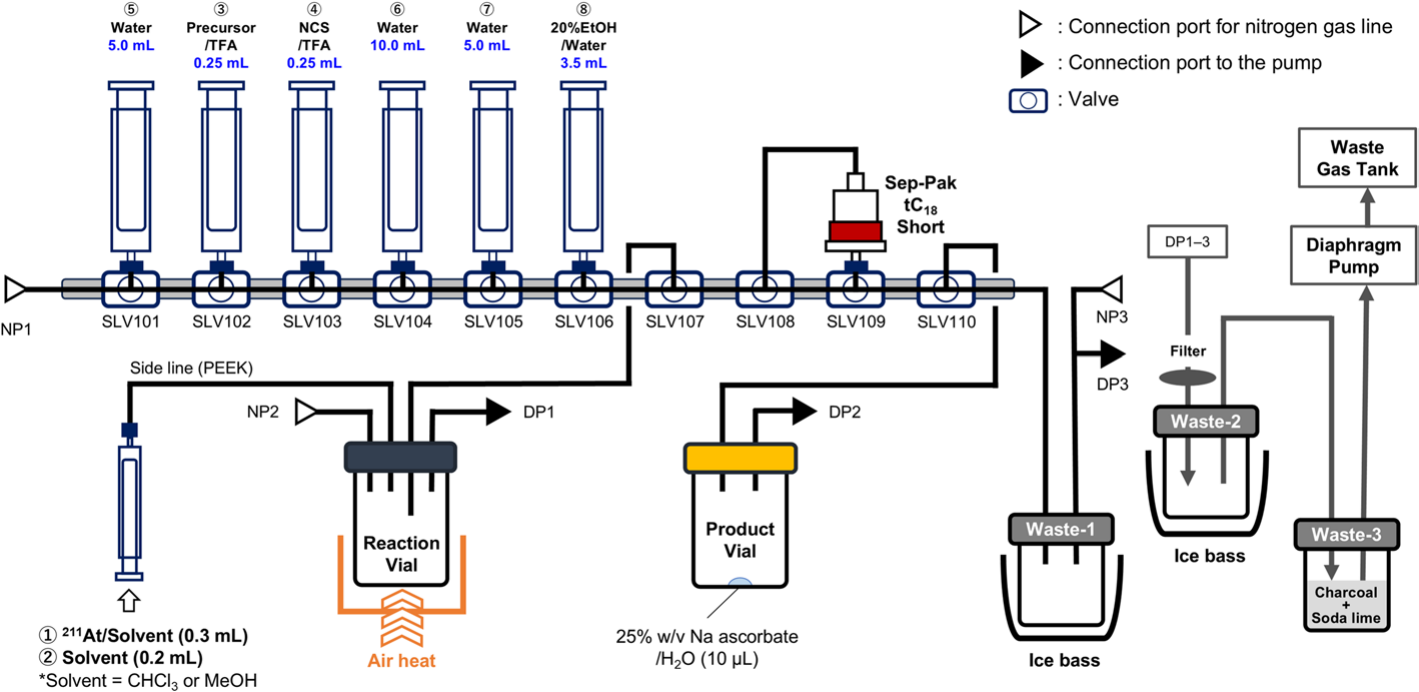
Schematic of the automated production system for [^211^At]MABG

[^211^At]MABG was synthesised via an astatodesilylation reaction using an aryl silyl precursor (Scheme 1) and a general-purpose automated radiosynthesiser, COSMiC-Mini (NMP Business Support Co., Ltd., Hyogo, Japan) (Song et al. 2021; Liu et al. 2023; Iida et al. 2023), described in Fig. 3. Prior to initiating the automatic sequence, the reagents were prepared; the TFA solution of the reaction reagents, sterile water, and 20% EtOH/H_2_O were loaded into disposable syringes and attached to ports SLV101-106. An SPE column (Sep-Pak tC_18_ Plus Short; Waters Corp., MA, USA) was washed with EtOH and 20% EtOH/H_2_O, conditioned with sterile water, and attached to SLV108 and SLV109. Subsequently, 10 μL of 25% w/v sodium ascorbate/H_2_O was added into the product recovery vial as a stabilizer, and was attached to SLV110 and DP2. Four lines were inserted into the reaction vial and connected to NP2, DP1, and SLV107. For syntheses using ^211^At in MeOH, potassium carbonate (K_2_CO_3_; 72 μmol) was added to the reaction vial. Following system setup, the automation synthesis program was initiated. A CHCl_3_ or MeOH solution of ^211^At (44.6–586.1 MBq, 100–500 μL) was introduced to the reaction vial through the sideline (polyetheretherketone, PEEK) using a disposable syringe. Furthermore, CHCl_3_ or MeOH (200 μL) was added to flush the line. The solvent was then evaporated under a nitrogen gas flow at 40 °C for 3 min (CHCl_3_) or 5 min (MeOH). After cooling to room temperature, the TFA solution of *m*TMSBG (1.0 μmol/250 μL) was automatically added from a disposable syringe attached to SLV102, followed by the TFA solution of *N*-chlorosuccinimide (NCS; 2.0 μmol/250 μL) from SLV103. The reaction mixture was heated to 70 °C for 10 min using an integrated air heater, then allowed to cool below 40 °C. The line was rinsed with sterile water (5 mL) from SLV101, followed by the addition of sterile water (10 mL) from SLV104 into the reaction vial. The resulting aqueous solution was transferred onto the SPE column, after which an additional 5 mL of sterile water from SLV105 was added to the reaction vial and passed through the SPE column. Finally, 20% EtOH/H_2_O (3.5 mL) was eluted through the SPE column from SLV106 into the product vial containing 25% w/v sodium ascorbate in H_2_O (10 μL). The line was purged with nitrogen gas and the collected solution was concentrated at 40 °C for 3 min using a smart evaporator. The obtained solution was analysed via radio-HPLC and the RCP (%) was determined by the peak area ratio of the radio-chromatogram. The radiochemical yield (RCY) was calculated as follows:

$${\text{RCY}}\left( \% \right) = \left[ {\left( {{\text{activity}}\;{\text{of}}\;{\text{eluted}}\left[ {^{{{211}}} {\text{At}}} \right]{\text{MABG}}\;{\text{from}}\;{\text{SPE}}\;{\text{column}}} \right)/\left( {{\text{activity}}\;{\text{of}}\;^{{{211}}} {\text{At}}\;{\text{used}}\;{\text{for}}\;{\text{reaction}}} \right)} \right] \times {1}00.$$

The [^211^At]MABG peak was identified based on the retention time of *m*-iodobenzylguanidine (MIBG) as a reference compound. Upon completion, the automated synthesis system was disassembled, and the residual radioactivity was quantified in the liquid delivery lines, waste bottles (Waste-1, -2, and -3), filter, reaction vial, and SPE column, to assess the distribution of unreacted ^211^At.

### Radiopharmaceutical preparation

A Millex-FG filter unit (0.20 μm, PTFE, 25 mm; Merck Millipore Ltd.) and Millex-GV filter unit (0.22 μm, PVDF, 33 mm; Merck Millipore Ltd.) were connected to a 50-mL sterile vial with injection needles. Saline (6 mL) was added to the obtained product solution using a sterile disposable syringe, and the diluted product solution was drawn into the syringe and transferred into the sterile vial through the GV filter. This procedure was repeated with an additional 10 mL of saline. Subsequently, 20 mL of sterile saline was introduced into the vial through the GV filter. Portions of the final sterile solution (2 and 1 mL) were sampled into a sterile disposable syringe and GC glass vial, respectively, for analysis via GC and HPLC to assess residual solvents and determine RCP.

The pH of the final sterile solution was measured using a benchtop pH meter F-74 (HORIBA, Ltd, Kyoto, Japan), calibrated using standard pH buffer solutions.

Endotoxin testing was conducted using the turbidimetric method with a Toxinometer® ET-6000 (FUJIFILM Wako Pure Chemical, Tokyo, Japan) in accordance with the Japanese Pharmacopoeia guidelines. Based on a maximum dose of 40 mL, the acceptance criterion was set at 3.75 EU/mL, and the [^211^At]MABG solution was diluted tenfold.

Sterility testing was performed by direct inoculation into two different culture media; 0.5 mL of the [^211^At]MABG solution was added to 10 mL of fluid thioglycollate medium and soybean casein digest medium, respectively. The samples were incubated at 32.5 and 22.5 °C using a double chamber incubator IQ822 (Yamato Scientific Co., Ltd, Tokyo, Japan), and the microbial growth was assessed on day 14. Method suitability testing confirmed microbial growth in the presence of the [^211^At]MABG solution and positive control for all six strains listed in the Japanese Pharmacopoeia (*Bacillus subtilis*, *Candida albicans*, and *Aspergillus brasiliensis* in soybean casein digest medium; *Pseudomonas aeruginosa*, *Staphylococcus aureus*, and *Clostridium sporogenes* in fluid thioglycollate medium) within 3 or 5 day.

A filter integrity test was conducted to evaluate the performance of the sterilised filter. The bubble point pressure of the aqueous solution, as specified by the manufacturer (> 345 kPa), was used as the acceptance criterion and was measured using a UG-FT02 filter integrity tester (Universal Giken, Kanagawa, Japan).

## Results

### Production of ^211^At

**Fig. 4:**
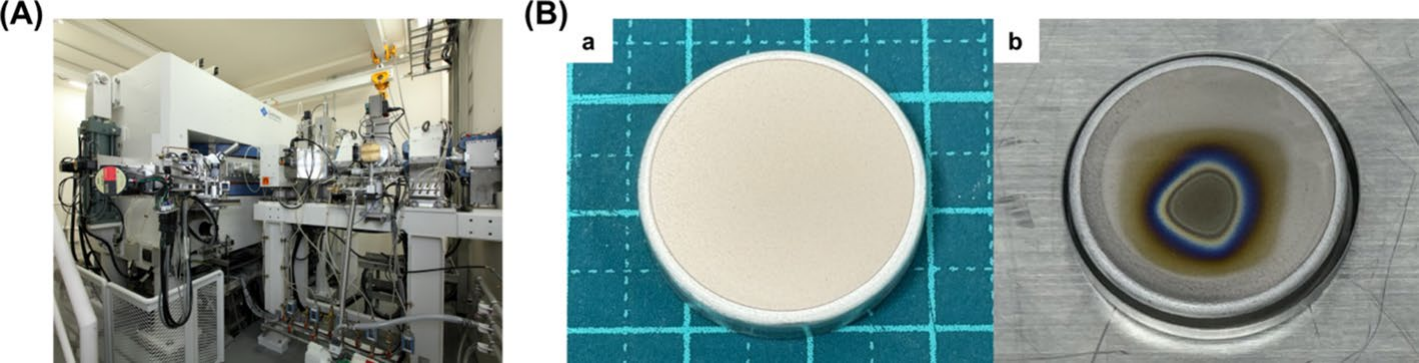
**A** MP-30 accelerator and **B** target of ^209^Bi plate (a) before and (b) after irradiation

Astatine-211 (^211^At) was produced via the ^209^Bi(α,2n)^211^At reaction using the MP 30 accelerator (Fig. 4A). The ^209^Bi target plate was prepared by physical vapor deposition onto an aluminium plate and irradiated with 28.6 MeV alpha particles at a beam current of 20 µA. No visible melting or deformation of the ^209^Bi target was observed following irradiation (Fig. 4B).

The theoretical TTY of the ^209^Bi(α,2n)^211^At reaction under the aforementioned irradiation conditions was 28.7 MBq/μA·h (International Atomic Energy Agency (IAEA)). The experimentally determined TTY was 27.4 ± 0.8 MBq/μA·h, corresponding to 95.5% of the theoretical value. The recovery yield of ^211^At following dry purification was 65.1 ± 5.0% (n = 14) at EOB, and the working time required from placing the irradiated target in the dry distillation system to obtaining the ^211^At solution was 25–30 min. γ-Ray spectrometry of the isolated ^211^At solution revealed characteristic peaks corresponding to ^211^At (687.00 keV), ^211^Po (897.80 and 569.65 keV), and ^207^Bi (569.70 keV) (Fig. S1). Notably, no peaks deriving from ^210^At (1181.40 keV) and ^210^Po (803.06 keV) were detected.

### Automation synthesis of [^211^At]MABG

**Fig. 5:**
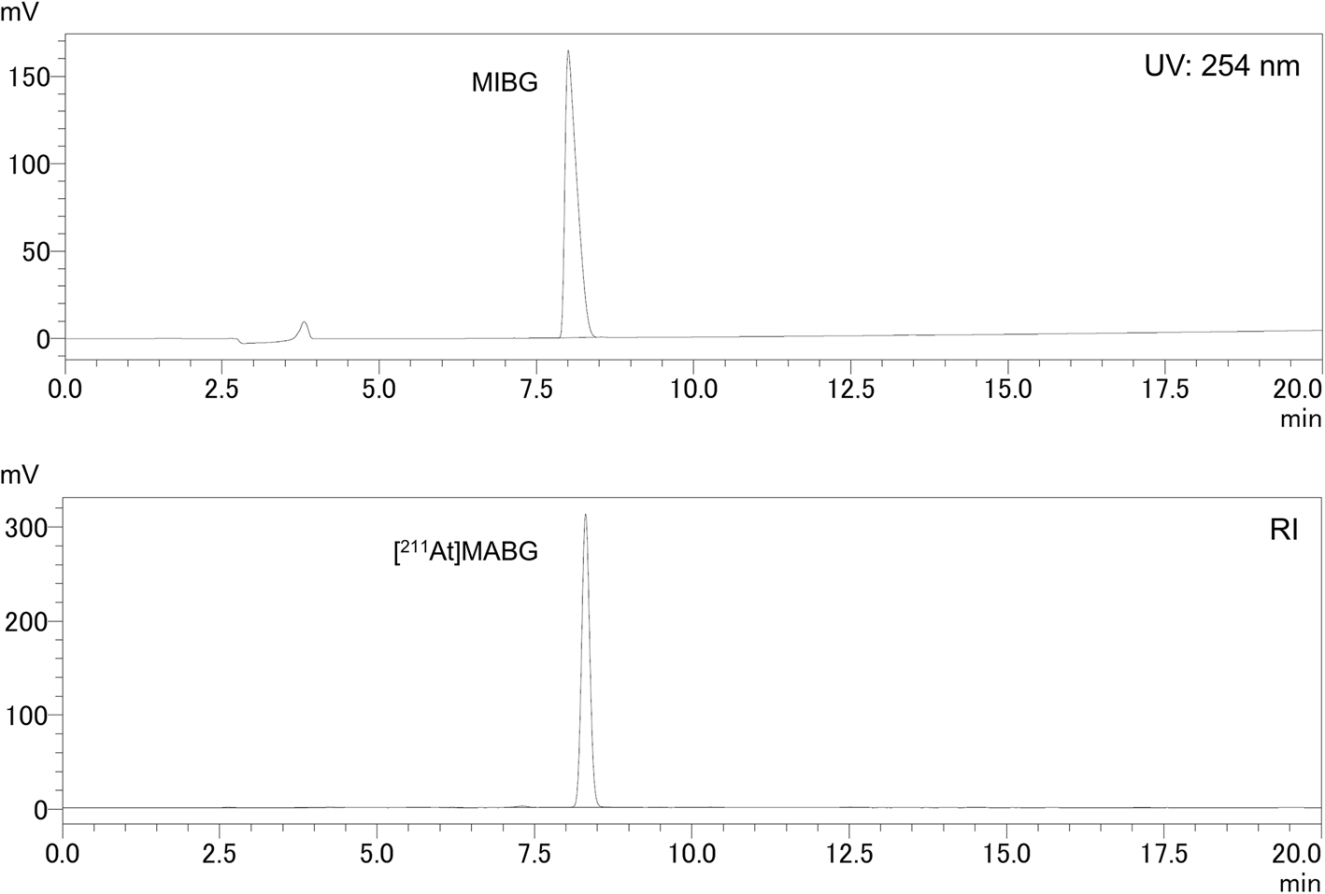
HPLC chromatogram of MIBG (authentic sample) and [^211^At]MABG

Table 1 summarizes the synthesis outcomes obtained in this study. Entries 1–6 correspond to experiments conducted using CHCl_3_ as the elution solvent, yielding RCYs of 80.3 ± 4.4% (decay-corrected RCY: 84.0 ± 4.5%, n = 6). In entries 7–10, MeOH was employed as the elution solvent and potassium carbonate was added; however, its effect was minimal, with RCYs showing no significant change (RCY: 72.6 ± 13.6%, decay-corrected RCY: 76.1 ± 14.3%, n = 4). Across all tested conditions (Entries 1–10), RCP exceeded 98% and the automated synthesis of [^211^At]MABG was completed within 33 min. Due to the absence of stable astatine isotopes, identification of [^211^At]MABG was based on the retention time of MIBG as a reference compound. As shown in Fig. 5, the retention time of [^211^At]MABG was 8.3 min, while that of MIBG (authentic sample) was 8.0 min. The significant decrease in RCY is typically caused by radiolysis, being effectively suppressed by the addition of sodium ascorbate.

**Table 1:** Reaction scale and radiochemical yield and purity of [^211^At]MABG

Entry	Organic solution used to elute ^211^At	Activity of ^211^At used for reaction (MBq)	RCY (%)	Decay-corrected RCY (%)	RCP (%)	Automated synthesis time (min)
1	CHCl_3_	66.9	86.8	91.0	99.7	29
2		49.2	81.7	85.3	98.9	26
3		44.6	74.0	78.0	99.0	33
4		289.8	79.4	82.6	99.2	25
5		563.5	77.6	81.2	97.8	28
6		586.1	82.1	85.9	99.3	28
7	MeOH	54.8	80.7	84.7	99.9 >	30
8		48.3	52.6	55.0	99.9 >	27
9		46.4	75.6	79.2	99.7	29
10		252.0	81.5	85.4	98.3	29

Radiochemical yield (RCY; %) = [(activity of eluted [^211^At]MABG from SPE column)/(activity of ^211^At used for reaction)] × 100. Radiochemical purity (RCP; %) was determined via radio-HPLC analysis. MeOH was concentrated in the presence of K_2_CO_3_

Following synthesis, the system was disassembled and the radioactivity of the delivery lines, waste bottles, filter, reaction vial, and column was measured. The majority of residual ^211^At was observed in the SPE column (8.4 ± 4.1%; n = 10) and the side line (2.8 ± 2.6%; n = 10). The radioactivity detected in Waste-1, which collected the waste solution, was 1.2 ± 1.0% (n = 10). The low levels of radioactivity observed in Waste-2 (1.6 ± 1.7%; n = 10) and Waste-3 (1.0 ± 0.9%; n = 10), both connected to the exhaust line, indicated negligeable volatilization losses of ^211^At during synthesis.

### Radiopharmaceutical preparation

**Fig. 6:**
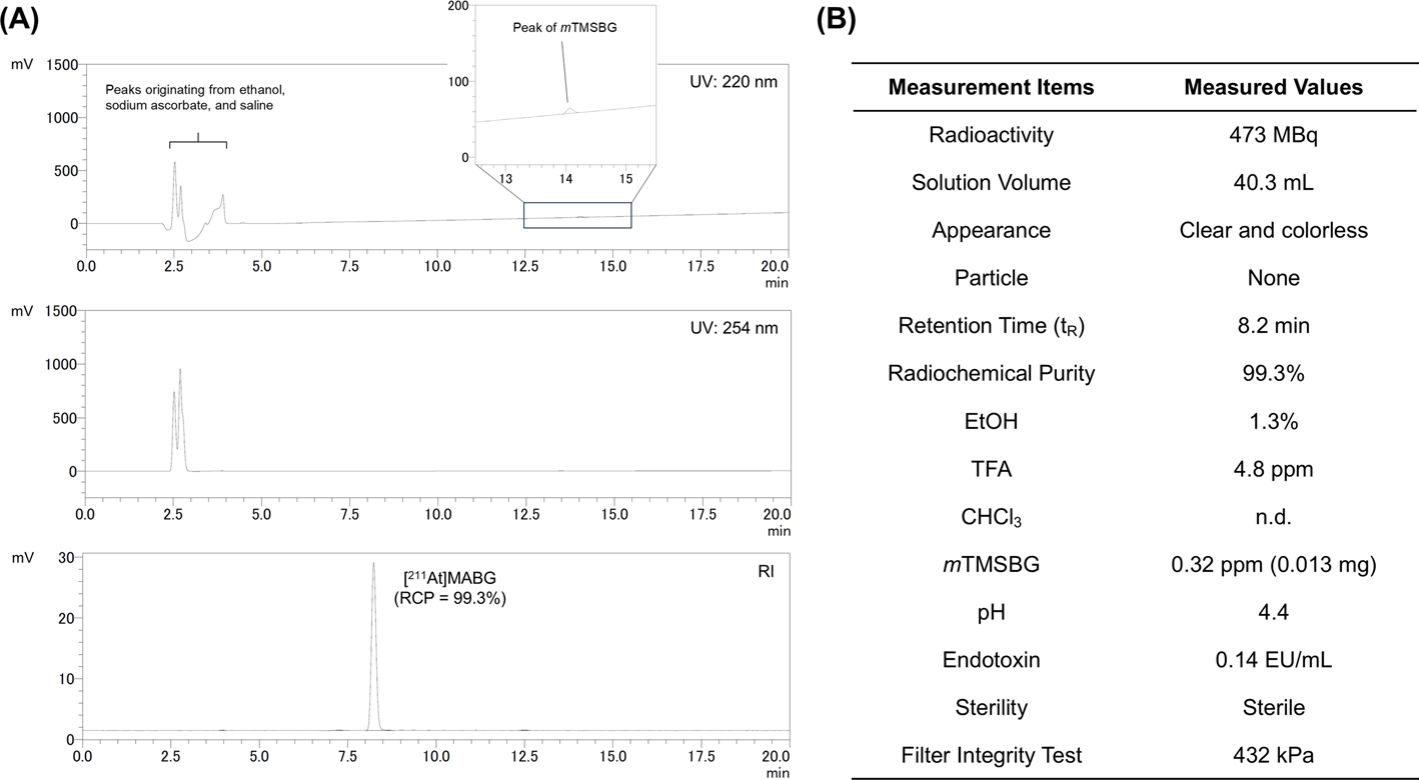
**A** UV and radio-HPLC chromatograms of the [^211^At]MABG solution after sterile filtration, and **B** measurement items and measured values. *Note: The HPLC chromatogram of physiological saline containing ethanol and sodium ascorbate is provided in Fig. S4 of the Supporting Information

The [^211^At]MABG solution was diluted with saline and passed through a sterile Millex-GV filter; minimal adsorption of [^211^At]MABG was observed on the filter. The RCP value of [^211^At]MABG in the final sterile solution was > 99% (Fig. 6), with no evidence of degradation of [^211^At]MABG during dilution or filtration. Furthermore, GC and HPLC analyses confirmed that the EtOH content was 1.3%, the residual TFA level was 4.8 ppm, and undetectable levels of residual CHCl_3_ (below the detection limit). A minor UV peak corresponding to the precursor (*m*TMSBG) was observed at 14.1 min (Fig. 6A). The pH of the final [^211^At]MABG sterile solution was 4.4. The endotoxin concentration was 0.14 EU/mL, and sterility tests showed no microbial growth. The bubble-point value for the filter integrity test was 432 kPa (≥ 345 kPa). γ-Ray spectrometry of the [^211^At]MABG solution confirmed characteristic peaks for ^211^At (687.00 keV) and ^211^Po (897.80 and 569.65 keV) (Fig. S2).

## Discussion

During the production of ^211^At via the ^209^Bi(α,2n)^211^At reaction, it is essential to maintain the energy of the α-beam below the threshold (28.6 MeV) for the production of ^210^At in the (α,3n) reaction (Morzenti et al. 2018). ^210^At undergoes β^+^ decay with a half-life of 8.1 h, yielding ^210^Po, a toxic isotope with a longer half-life (138.4 d) compared with ^211^Po (0.516 s). It is therefore crucial to suppress the production of ^210^At and ensure the radionuclidic purity of ^211^At. In this study, no peaks corresponding to ^210^At were detected in the γ-ray spectrum of the purified ^211^At solution.

^211^At was purified using a dry distillation system and was recovered from the Teflon tubing using a volatile solvent, thereby minimizing the impact of the recovery solution on subsequent astatination reactions. CHCl_3_ and MeOH have been previously employed as elution solvents at the Advanced Clinical Research Center, based on reports regarding the loss of volatile astatine during the concentration process. CHCl_3_ has been shown to reduce astatine loss during solvent concentration (Aneheim et al. 2019). Furthermore, our previous study demonstrated that when MeOH is used, coexisting alkaline metal salts such as K_2_CO_3_, can similarly mitigate astatine loss during solvent concentration (Tanaka et al. 2021). In this study, both approaches were integrated into the automated synthesis system for [^211^At]MABG, and their RCYs were compared. As a result, optimization of the synthetic process enabled high-yield production in both cases [decay-corrected RCY: 84.0 ± 4.5% (CH_3_Cl) vs 76.1 ± 14.3% (MeOH)]. However, the ~ 20% points decrease in RCY observed in a preliminary experiment (Entry 8, Table 1) was attributed to insufficient interaction between K_2_CO_3_ and the MeOH solution of ^211^At prior to the concentration step. Considering reproducibility and simplicity, CHCl_3_ was selected as the preferred eluent in this study. Nonetheless, the feasibility of employing MeOH as the eluent with K_2_CO_3_ during the concentration process warrants attention from both academic relevance and practical applicability.

The liquid transfer in COSMiC-mini is regulated by adjusting the pressure within vials or bottles using a diaphragm pump and nitrogen gas flow. During the addition of the reaction reagents and the dilution process, rapid liquid transfer did not affect the reaction yield. However, transferring the diluted solution to the SPE column required a precisely controlled flow rate. Preliminary experiments indicated that high flow rates of the diluted reaction solution to the SPE column significantly decreased the retention of [^211^At]MABG on the column. Therefore, to enhance the retention of [^211^At]MABG on the SPE column, the pressure in the reaction vial was gradually increased using nitrogen gas, while the pressure in the Waste-1 bottle was gradually reduced using a vacuum pump during solution transfer steps.

The decomposition of radiopharmaceuticals via radiolysis leads to decreased RCPs. Reactive species such as hydroxyl radicals are generated through the radiolysis of water, induced by the ionizing effects of radiation. These species contribute to the degradation of radiopharmaceuticals. Specifically, as the radioactive concentration of [^211^At]MABG increases, the production of reactive species is enhanced, resulting in decomposition of the compound. Therefore, the addition of a reducing radical scavenger (e.g. sodium ascorbate) is essential for maintaining the stability of [^211^At]MABG during synthesis. Figure S3 (Supplementary Information) presents preliminary data from our facility evaluating the inhibitory effect of sodium ascorbate on the degradation of [^211^At]MABG. The stability of [^211^At]MABG (113.1 MBq/2 mL) was assessed under two conditions: without additives and with sodium ascorbate. The results clearly demonstrate that sodium ascorbate effectively suppresses the degradation of [^211^At]MABG. Based on these preliminary findings, a sodium ascorbate solution was preloaded into the product vials in the experimental setups for entries 1–10 (Table 1). Even at a high activity level of 586.1 MBq, no degradation of [^211^At]MABG was observed after 24 h. Conversely, in the absence of sodium ascorbate, an increase in the peak area at 7 min was observed over time. The peak corresponding to free ^211^At appeared before 5 min, suggesting that ^211^At was not cleaved from the aromatic ring. Instead, the guanidino moiety of [^211^At]MABG underwent degradation. However, structural characterization of the specific degradation products was not achieved in this study.

Vaidyanathan et al. reported a high-level synthesis of [^211^At]MABG using a tin precursor immobilized on a solid support. The precursor undergoes astatination upon treatment with a methanolic solution of ^211^At in the presence of acetic acid and hydroxy peroxide (Vaidyanathan et al. 2007b). [^211^At]MABG is subsequently isolated using C_18_ SPE or a cation exchange resin cartridge. The C_18_-based approach achieved RCYs of 63 ± 13%, but suffered from ~ 50% activity loss during methanol evaporation—possibly due to radiolysis, which, based on our findings, could potentially be mitigated by sodium ascorbate. The alternative isolation strategy using a cation exchange resin cartridge maintained comparable RCYs (63 ± 9%; from initial ^211^At activity 14.4–658.6 MBq), with > 90% RCPs. The total synthesis time was ~ 120 min for the C_18_ method and ~ 70 min for the cation exchange method.

In this study, [^211^At]MABG was synthesized with high yields (RCY 80.3 ± 4.4%; decay-corrected RCY 84.0 ± 4.5%; from an initial ^211^At activity of 44.6–586.1 MBq; Entries 1–6) and RCP (99.0 ± 0.7%; Entries 1–6), surpassing previously reported methods. Furthermore, the synthesis time was significantly reduced to 28.2 ± 2.8 min (Entries 1–6), and the entire process, including final formulation, was completed within 1 h. Notably, even at high radioactivity levels (563.5–586.1 MBq), a high RCY was maintained (RCY 77.6–82.1%; decay-corrected RCY 81.2–85.9%; Entries 5 and 6), enabling the production of 437–473 MBq of [^211^At]MABG in a single synthesis. Even though the quality of the [^211^At]MABG produced by the established methodology is comparable to the conventionally synthesized material, further in vivo studies are required to evaluate biodistribution, therapeutic efficacy, and toxicity. Comparative in vivo studies using mouse models are currently underway. Furthermore, the use of SPE for purification necessitates careful monitoring for the presence of trace amounts of the precursor in the final formulation, which constitutes an important parameter in toxicity evaluations. Looking ahead, we aim to explore the possibility of reducing the amount of precursor employed in the reaction, provided it does not compromise reaction efficiency.

Considering the reaction yield, synthesis time, and availability of precursors, the automated astatodesilylation method using COSMiC-Mini presents an efficient approach for [^211^At]MABG production. Furthermore, the programmable nature of COSMiC-Mini allows on-site customisation, facilitating the adaptation of this protocol for the synthesis of other ^211^At-labeled radiopharmaceuticals. Therefore, the manufacturing techniques and expertise described in this study offer a valuable tool for the future production of ^211^At-labeled radiopharmaceuticals.

## Conclusions

In this study, a robust methodology was established for the clinical-scale production of [^211^At]MABG. The process encompasses the production of ^211^At via the ^209^Bi(α,2n)^211^At reaction using an MP-30 cyclotron, followed by purification via dry distillation with the At-HDS100 system, and automated radiosynthesis of [^211^At]MABG using the COSMiC-mini. The automated synthesis consistently yielded [^211^At]MABG with high radiochemical yields (RCYs: 80.3 ± 4.4%; decay-corrected RCY: 84.0 ± 4.5%) and radiochemical purity (RCP: 99.0 ± 0.7%) within a short synthesis time of 28.2 ± 2.8 min. Notably, the addition of sodium ascorbate significantly enhanced the radiolytic stability of [^211^At]MABG, contributing to the reproducibility and reliability of the method. The final product was obtained as a sterile, injectable solution that meets standard physicochemical and radiochemical quality requirements. This integrated approach represents a significant advancement toward the clinical translation of [^211^At]MABG as a next-generation targeted alpha therapy.

## Supplementary Information

Supplementary material 1.

## Data Availability

All data generated or analysed during this study are included in this published article and its supplementary information files.

## Abbreviations

CHCl_3_: Chloroform
EOB: End of bombardment
FID: Flame ionization detector
FKM: Fluoroelastomer
GC: Gas chromatography
HPLC: High performance liquid chromatography
ICP-MS: Inductively coupled plasma mass spectrometry
K_2_CO_3_: Potassium carbonate
MABG: *m*-Astatobenzylguanidine
MeOH: Methanol
MIBG: *m*-Iodobenzylguanidine
*m*TMSBG: 1-(3-(Trimethylsilyl)benzyl)guanidine
NCS: *N*-Chlorosuccinimide
NET: Neuroendocrine tumour
PEEK: Polyetheretherketone
PTFE: Polytetrafluoroethylene
PVDF: Polyvinylidene fluoride
RCP: Radiochemical purity
RCY: Radiochemical yield
SPE: Solid-phase extraction
TFA: Trifluoracetic acid
TTY: Thick target yield
UV: Ultraviolet
