# Supplementary material for: Production of 211At and automated radiosynthesis of [211At]MABG via electrophilic astatodesilylation
Source: EJNMMI Radiopharm Chem. 2025 Aug 5;10:52. doi: 10.1186/s41181-025-00376-1 (PMC12325123; doi:10.1186/s41181-025-00376-1)
Supplement: Supplementary file 1 — Supplementary material 1. [file 41181_2025_376_MOESM1_ESM.pdf]

SUPPORTING INFORMATION

Production of ^{211}At and Automated Radiosynthesis of [^{211}At]MABG *via* Electrophilic Astatodesilylation

Yuto Kondo,[a] Taiki Joho,[a] Shigenori Sasaki,[a,b] Kazumasa Mochizuki,[a,b] Naoko Hasegawa,[a] Naoyuki Ukon,[a] Ken-ichi Nishijima,[a] Kohshin Washiyama,[a] Hiroshi Tanaka,[c,d] Tatsuya Higashi,[e] Noriko S. Ishioka,[f] Kazuhiro Takahashi[a,]*

a. Advanced Clinical Research Center, Fukushima Global Medical Science Center, Fukushima Medical University, 1 Hikarigaoka, Fukushima 960-1295, Japan.

b. SHI Accelerator Service Ltd., 7-1-1 Nishigotanda, Shinagawa, Tokyo, 141-0031, Japan.

c. Department of Chemical Science and Engineering, Tokyo Institute of Technology, 12-12-1-H101 Ookayama, Meguro, Tokyo 152-8552, Japan.

d. Laboratory of Pharmaceutical Chemistry, Juntendo University, 6-8-1 Hinode, Urayasu, Chiba 279-0013, Japan.

e. Department of Molecular Imaging and Theranostics, Institute for Quantum Medical Science, National Institutes for Quantum Science and Technology, 4-9-1, Anagawa, Inage, Chiba-City, Chiba, 263-8555, Japan.

f. Department of Quantum-Applied Biosciences, Takasaki Institute for Advanced Quantum Science, National Institutes for Quantum Science and Technology, 1233 Watanuki, Takasaki, Gunma 370-1292, Japan.

*Corresponding author: Kazuhiro Takahashi

e-mail: ktakahas@fmu.ac.jp

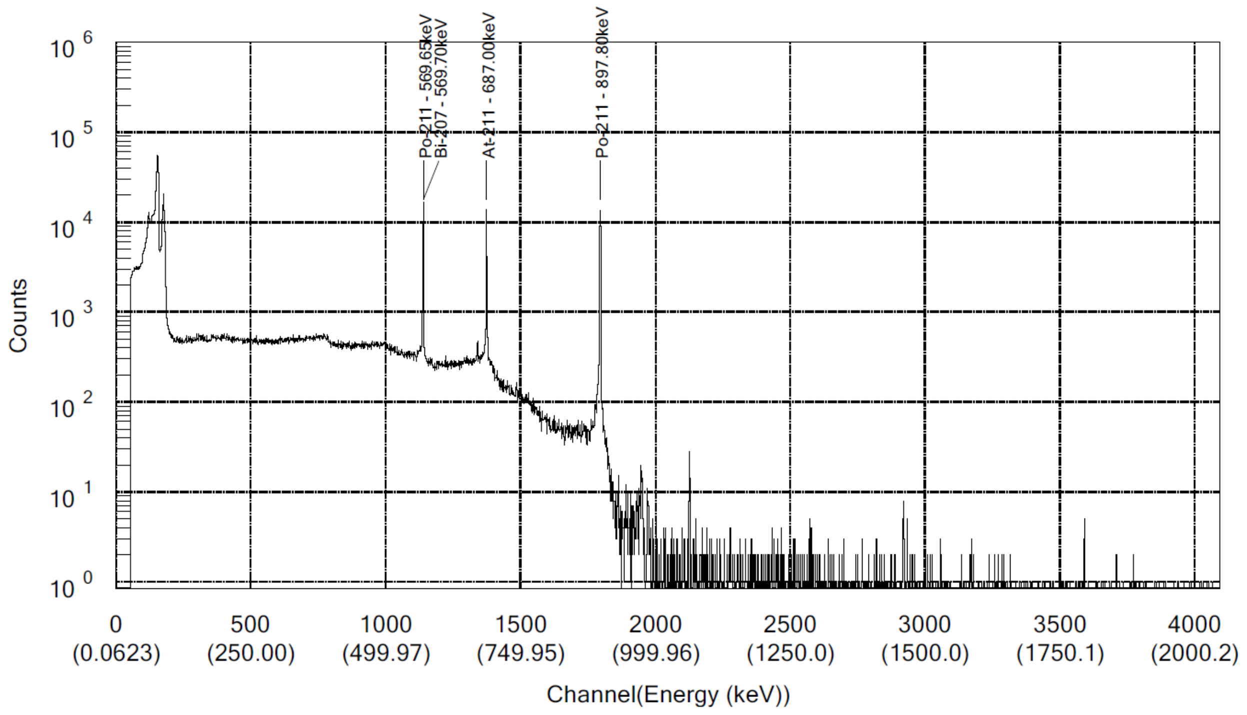

Figure S1. Typical γ-ray spectrum of the ^{211}At chloroform solution after purification.

γ-Rays were observed for ^{211}At (687.00 keV), ^{211}Po (897.80 and 569.65 keV), and ^{207}Bi (569.70 keV). The sample was placed 50 cm from the detector and an 8-mm-thick lead shield was inserted between the sample and the detector.

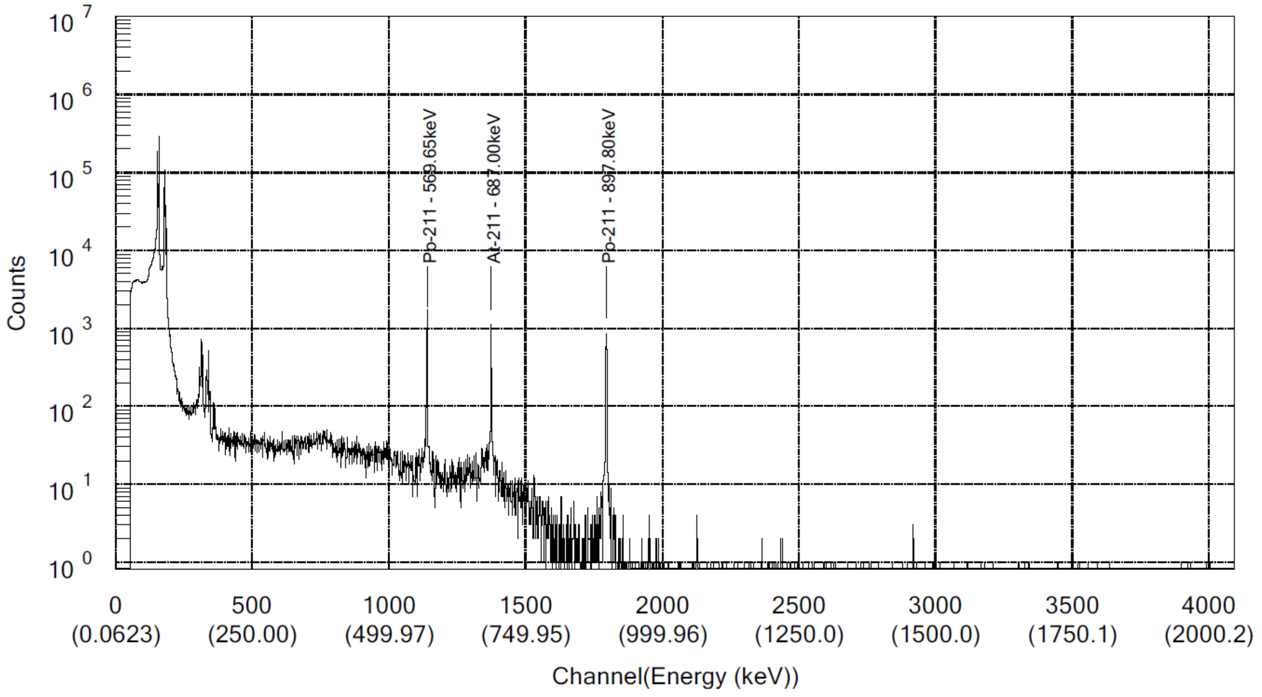

Figure S2. Typical γ-ray spectrum of the [^{211}At]MABG solution after purification.

γ-Rays were detected for ^{211}At (687.00 keV) and ^{211}Po (897.80 and 569.65 keV). The spectrum was acquired with the sample placed 50 cm from the detector.

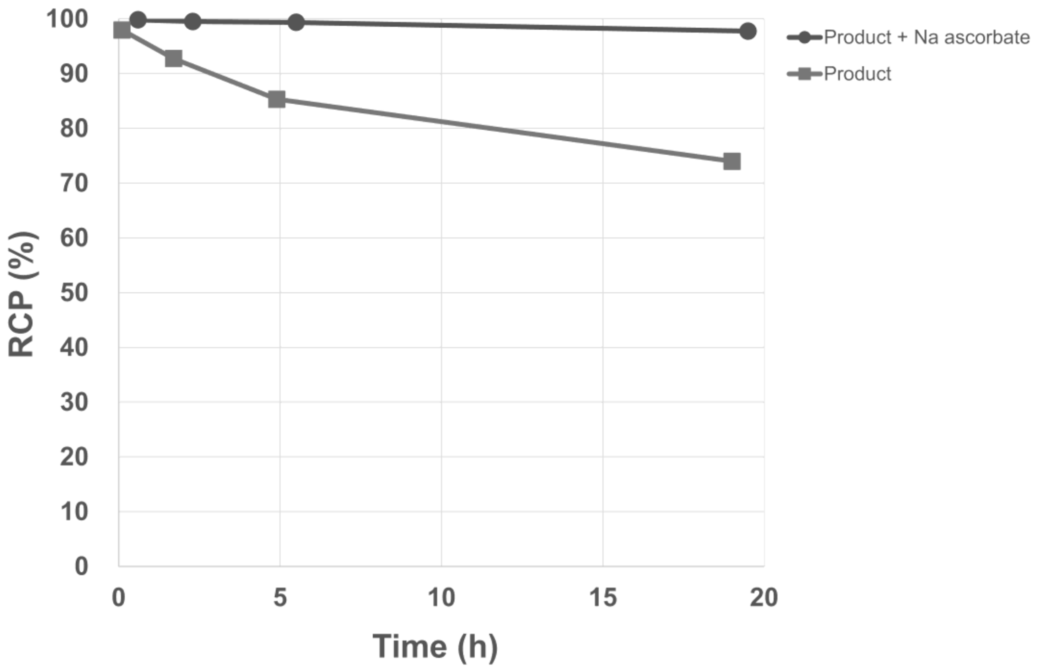

Figure S3. Effect of sodium ascorbate on the prevention of radiolytic decomposition of [^{211}At]MABG.

RCP (%) was determined via radio-HPLC analysis.

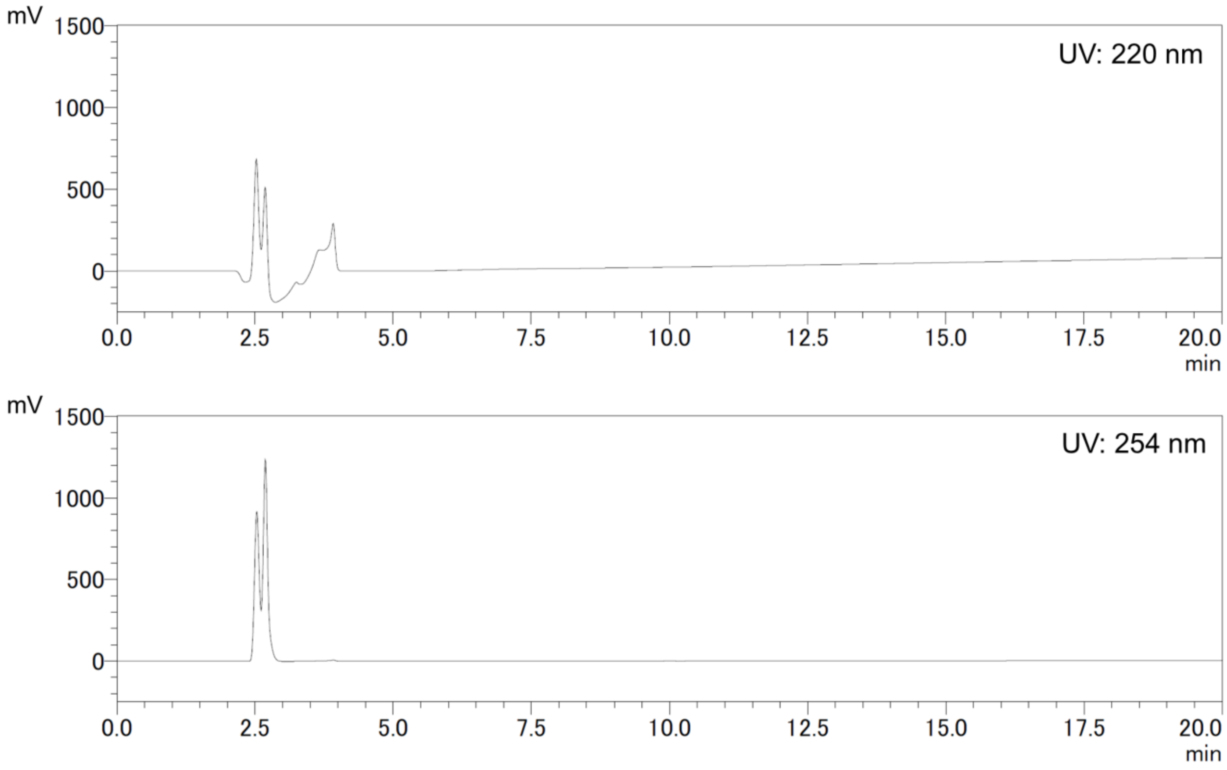

Figure S4. UV-HPLC chromatogram of saline containing ethanol and sodium ascorbate.
